# Mitochondrial Dysfunction and Impaired Antioxidant Responses in Retinal Pigment Epithelial Cells Derived from a Patient with *RCBTB1*-Associated Retinopathy

**DOI:** 10.3390/cells12101358

**Published:** 2023-05-10

**Authors:** Zhiqin Huang, Dan Zhang, Shang-Chih Chen, Di Huang, David Mackey, Fred K. Chen, Samuel McLenachan

**Affiliations:** 1 Centre for Ophthalmology and Visual Science, The University of Western Australia, Crawley, WA 6009, Australia; zhiqin.huang@lei.org.au (Z.H.);; 2 Lions Eye Institute, Nedlands, WA 6009, Australia; 3 Department of Ophthalmology, Royal Perth Hospital, Perth, WA 6000, Australia; 4 Ophthalmology, Department of Surgery, The University of Melbourne, Parkville, VIC 3010, Australia

**Keywords:** RCBTB1, inherited retinal disease, retinal pigment epithelium, oxidative stress, mitochondria

## Abstract

Mutations in the *RCBTB1* gene cause inherited retinal disease; however, the pathogenic mechanisms associated with RCBTB1 deficiency remain poorly understood. Here, we investigated the effect of RCBTB1 deficiency on mitochondria and oxidative stress responses in induced pluripotent stem cell (iPSC)-derived retinal pigment epithelial (RPE) cells from control subjects and a patient with *RCBTB1*-associated retinopathy. Oxidative stress was induced with tert-butyl hydroperoxide (tBHP). RPE cells were characterized by immunostaining, transmission electron microscopy (TEM), CellROX assay, MitoTracker assay, quantitative PCR and immunoprecipitation assay. Patient-derived RPE cells displayed abnormal mitochondrial ultrastructure and reduced MitoTracker fluorescence compared with controls. Patient RPE cells displayed increased levels of reactive oxygen species (ROS) and were more sensitive to tBHP-induced ROS generation than control RPE. Control RPE upregulated *RCBTB1* and *NFE2L2* expression in response to tBHP treatment; however, this response was highly attenuated in patient RPE. RCBTB1 was co-immunoprecipitated from control RPE protein lysates by antibodies for either UBE2E3 or CUL3. Together, these results demonstrate that *RCBTB1* deficiency in patient-derived RPE cells is associated with mitochondrial damage, increased oxidative stress and an attenuated oxidative stress response.

## 1. Introduction

Mutations in the *RCC1 and BTB domain-containing protein 1* (*RCBTB1*) gene have been reported to cause a spectrum of inherited retinal diseases (IRDs) [1,2,3,4,5]. To date, fifteen cases from eleven unrelated families have been reported to be associated with biallelic variants in the *RCBTB1* gene; however, the pathogenic mechanisms associated with RCBTB1 deficiencies remain poorly understood. We previously described the natural history of chorioretinal atrophy progression of a patient with compound heterozygous *RCBTB1* mutations (c.170delG and c.707delA) over 5 years [3]. Consistent with our previous optical coherence tomography (OCT) findings, which demonstrated diffuse attenuation and irregularity of the retinal pigment epithelium (RPE) layer and the adjacent ellipsoid zone in the proband, a multimodal imaging study reported by Catomeris et al. also demonstrated widespread RPE irregularity and progressive RPE atrophy in patients carrying biallelic *RCBTB1* variants [5]. Collectively, clinical observations in *RCBTB1*-associated retinopathy suggest that the primary lesion is most likely located within the RPE layer, followed by secondary photoreceptor cell apoptosis. Recent recognition of the phenotypic similarity between RCBTB1-associated retinopathy and the most severe forms of mitochondrial retinopathy [6] further suggests RPE mitochondria may play a role in disease pathogenesis.

To study the effects of RCBTB1 deficiency in human RPE cells, we previously generated induced pluripotent stem cell (iPSC) lines from a patient with *RCBTB1*-associated retinopathy caused by biallelic frameshifting mutations in the *RCBTB1* gene [7]. RPE generated from these iPSC lines displayed reduced expression of genes involved in the NFE2L2 oxidative stress pathway [8], supporting similar findings reported in lymphocytes isolated from patients with *RCBTB1*-associated retinopathy [2]. To further elucidate the effects of RCBTB1 deficiency in RPE cells, we characterized reactive oxygen species (ROS) generation and activation of *NFE2L2* expression in patient- and control-derived RPE cells under basal conditions and in response to an oxidative stress challenge with tert-butyl hydroperoxide (tBHP). Additionally, we characterized mitochondria in patient-derived RPE cells by electron microscopy and Mitotracker labelling.

## 2. Materials and Methods

### 2.1. Human-Induced Pluripotent Stem Cell Lines

The human iPSC lines used in this study were obtained with informed consent following protocols approved by the Human Research Ethics Committee (2001-053) at Sir Charles Gairdner Hospital (Nedlands, Western Australia, Australia) and the Human Ethics Office of Research Enterprise at The University of Western Australia (RA/4/1/7916). This study was performed in accordance with the Declaration of Helsinki. The protocols used in this study were approved by the Institutional Biosafety Committee of the Harry Perkins Institute of Medical Research, University of Western Australia (NLRD 02-2020). The clinical phenotype of the patient and generation of iPSC lines was reported previously [3,7].

### 2.2. RPE Differentiation and Cell Culture

RPE monolayers were generated from iPSC lines from a patient with RCBTB1-associated retinopathy and two healthy donors using our published methods [8]. Media were changed twice per week and RPE cells were passaged using TrypLE™ Express Enzyme (Thermo Fisher Scientific, Waltham, MA, USA) at a split ratio of 1:4. RPE cells were used at passage 3–4 for experiments 8 weeks after seeding. Expression of RPE markers in iPSC-derived RPE cells was characterized by immunofluorescence staining and quantitative PCR (Supplementary Figure S1), as previously described [8]. Primers and antibodies used for RPE characterisation are listed in Supplementary Tables S1 and S2, respectively.

### 2.3. Phagocytosis Assay

Bovine rod photoreceptor outer segment (POS) was purchased from InVision BioResources (Seattle, WA, USA). For POS labelling, fluorescein isothiocyanate (FITC) was added to the POS following the published procedure [9]. Phagocytosis assays were performed according to McLaren and Buskin et al.’s protocols [10,11]. The ratio of POS and iPSC-RPE cells was 10:1, and cells were incubated with POS for 6 h at 37 °C. For each RPE cell line, one well without POS was included as negative control, while total and internalized FITC-POS were assessed in triplicate wells. In all phagocytosis experiments, unbound POS were washed away with phosphate-buffered saline supplemented with 1 mM MgCl_2_ and 0.2 mM CaCl_2_ (PBS-CM) before fixation. To quantify internalized POS, cells were treated with 0.4% trypan blue and incubated at room temperature for 10 min to quench fluorescence of surface-bound FITC-labelled POS. For flow cytometry, cells were suspended in FACS buffer (PBS with 1% FBS and 1 mM EDTA) and transferred to FACS tubes. Samples were fixed and run on a BD LSRFortessa X-20 flow cytometer, and at least 5000 events were collected per sample. Untreated iPSC-RPE cells were used as negative control to set the live cell gate in each experiment. Data were analysed using FlowJo 10.4 software.

For the fluorescence microscopy method, cells were rinsed with PBS-CM following trypan blue incubation and fixed with 4% PFA for 10 min. Nuclei were stained with DAPI, and internalized phagocytosed POS were visualized using the Nikon A1 Confocal Laser microscope (Nikon, Tokyo, Japan). The number of internal POS per cell and per area was counted, and data analysis of differences was determined by unpaired student *t*-test.

### 2.4. Quantitative Real-Time PCR

RNA was harvested from RPE cells using Trizol (Thermo Fisher Scientific) following the manufacturer’s instructions. RPE cDNA was synthesized using the RT^2^ First Strand Kit (Qiagen, Hilden, Germany). Quantitative real-time PCR analysis (qRT-PCR) was conducted using the CFX Connect Real-Time System (BioRad, Hercules, CA, USA) with the RT^2^ SYBR Green qPCR Mastermix (Qiagen). Expression levels were normalized to *GAPDH* and expressed as mean fold-changes compared with untreated control RPE cells (*n* = 3 independently derived iPSC lines). Significance testing was performed using the student *t*-test, with *p* values < 0.05 considered significant. Primers used for qPCR are listed in Supplementary Table S1.

### 2.5. Transmission Electron Microscopy

For transmission electron microscopy (TEM), RPE cells were seeded on Millicell hanging cell culture inserts (24 well, 0.4 µm, Merck, Darmstadt, Germany) coated with Geltrex (Thermo Fisher Scientific). After 8 weeks of culture, inserts were removed and fixed with 2.5% glutaraldehyde (Grade I, Sigma-Aldrich, St. Louis, MO, USA) in 0.1 M phosphate buffer (pH 7.4) for 30 min at room temperature. The cells were secondarily fixed with 1% osmium tetroxide. Following dehydration using ethanol and acetone, RPE monolayers were embedded in resin in silicone rubber flat embedding moulds and cured for 48 h at 65 °C. Glass knives were made and mounted to a Leica EM UC6 ultramicrotome for ultrathin sectioning (80–120 nm). Sections were then collected onto copper grids and double stained with uranyl acetate (UA) and lead citrate. These sections were then visualized using a JEOL JEM-F200 transmission electron microscope (Jeol, Tokyo, Japan). Mitochondrial analysis was performed on TEM images collected from three wells of patient RPE and three wells of control RPE cells. Each well of RPE cells was derived from an independent, clonal iPSC line. Mitochondria length and area were measured in 6–13 TEM images per well using ImageJ and expressed as the mean values obtained from >200 mitochondria, with error bars indicating standard error of the mean (SEM). Statistical comparisons were performed using the unpaired *t*-test, with *p* values < 0.05 considered as significant.

### 2.6. MitoTracker Assay

The MitoTracker Orange CMTMRos Reagent (100 nM, ThermoFisher) was added to RPE cultures 8 weeks after seeding, and cells were incubated for 30 min, with or without 10 µM carbonyl cyanide chlorophenylhydrazone (CCCP, ThermoFisher). Following staining and fixation, cell fluorescence imaging was performed using the Olympus BX60 upright fluorescence microscope. Fluorescence signals in images were measured using ImageJ 1.53i (National Institute of Health, USA) and expressed as the mean values obtained from RPE cells generated from three independent iPSC lines, with error bars representing standard deviation. Statistical comparisons were performed using the unpaired *t*-test, with *p* values < 0.05 considered as significant.

### 2.7. CellROX Assay

CellROX Deep Red Reagent (Invitrogen, C10491) was used for measuring intracellular reactive oxidative species (ROS) levels. Healthy control and patient-derived iPSC-RPE cells were seeded in 96 well plates and cultured for 8 weeks. RPE cells were treated with tBHP at various concentrations (0 µM, 100 µM, 200 µM) for one hour, then stained with 5 µM CellROX^®^ Deep Red Reagent by adding the probe to the no phenol cell culture medium and incubating the cells for 30 min at 37 °C. Fluorescence signals were analysed on the CLARIOstar^®^ Plus microplate reader (BMG, Labtech, Ortenberg, Germany). The CellRox assay was performed in technical triplicates, and fluorescence signal data are expressed as the mean from three independent clonal iPSC lines, with error bars indicating standard deviation. The statistical analysis of differences was determined by unpaired *t*-test, with *p* values < 0.05 considered significant.

### 2.8. Immunoprecipitation Assay

Immunoprecipitation was carried out using the Immunoprecipitation kit (Abcam, Cambridge, United Kingdom, ab206996) as per the manufacturer’s instructions (Method B). For each reaction, 40 µL of protein A/G sepharose beads were washed twice with 1 mL wash buffer by slow-speed centrifugations and suspended as 50% slurry in 40 µL of wash buffer. Primary antibody was added into the slurry of protein A/G. Negative control beads without the addition of antibodies were also prepared. The antibody-bead mixture was incubated for 4 h at 4 °C on a shaker and was subsequently collected by slow-speed centrifugations. After cell lysis with ice-cold non-denaturing lysis buffer and protease inhibitor, protein quantification was performed using Quick Start™ Bradford 1× Dye Reagent (Bio-Rad). A total of 100 µg of cell lysate was added into the bead/antibody conjugate mixture and incubated under rotary agitation overnight at 4 °C. Beads were then pelleted and washed three times in 1× Wash Buffer. After the final wash step, beads were resuspended in 15 µL of 4× SDS-PAGE loading buffer without 2-mercaptoethanol and incubated at 50 °C for 5 min. Beads were pelleted, and the supernatant was collected and supplemented with 1 µL of 2-mercaptoethanol (elution 1). The bead pellet was then resuspended in 15 µL of 4X SDS-PAGE loading buffer with 2-mercaptoethanol and incubated at 50 °C for 5 min (elution 2). The eluted samples were boiled for 5 min and subjected to SDS-PAGE for electrophoresis and western blotting. Antibodies used are listed in Supplementary Table S2.

### 2.9. Western Blotting

Total protein was extracted using RIPA buffer (Sigma-Aldrich) and 1% Protease Inhibitor Cocktail (Sigma-Aldrich). Following centrifugation, protein lysates were collected, and the concentration was measured using the Quick Start™ Bradford Protein Assay (Biorad). Protein samples were loaded on 4–12% NuPAGE™ Bis-Tris Mini Protein Gels (ThermoFisher), and electrophoresis was conducted the XCell SureLock Mini-Cell Electrophoresis System (ThermoFisher). Protein was subsequently transferred to an Immobilon-FL PVDF membrane (Merck) using the Mini Protean II Trans-Blot Cell (Bio-Rad). Following transfer, membranes were cut at 50 kDa and 90 kDa to generate three pieces for staining with different antibodies. Membranes were blocked with 5% bovine serum albumin (BSA) in tris-buffered saline (TBS) for one hour at room temperature. The membrane was incubated in primary antibodies solution overnight at 4 °C, followed by incubation with secondary antibodies for 1.5 h at room temperature. Membranes were washed three times in TBS buffer supplemented with 0.1% TWEEN-20 and scanned using the Li-Cor Odyssey 9120 Infrared Imaging System (LI-COR Biosciences, Lincoln, NE, USA). Original fluorescence images of Western blots are shown in Supplementary Figure S4. Antibodies used are listed in Supplementary Table S2.

## 3. Results

### 3.1. RPE Differentiation

Expression of RPE markers was confirmed using immunocytochemistry and qRT-PCR in three control and three patient iPSC-derived RPE cell lines. Six weeks after seeding, iPSC-RPE cell cultures expressed RPE markers involved in the retinoid cycle (RPE65 and CRALBP), tight junction (ZO1), chloride channels (BEST1), phagocytosis function (MerTK), Na^+^/K^+^ ATPase, melanin biosynthesis (Tyrosinase) and transcription factors (PAX6 and MITF) (Supplementary Figure S1).

### 3.2. Photoreceptor Outer Segment Uptake in RCBTB1-Deficient RPE

To assess the ability of patient RPE cells to phagocytize POS, iPSC-RPE monolayers were incubated with FITC-labelled POS for six hours at a ratio of ten POS per cell. Analysis of the mean percentage of FITC-positive cells demonstrated no significant difference in phagocytosis function between control RPE and patient RPE (Supplementary Figure S2A). These findings were corroborated using a fluorescence microscopy method (Supplementary Figure S2B). Data analysis of the mean number of internal POS per cell and per area showed no significant changes between control and patient groups.

### 3.3. Ultrastructural Changes in RCBTB1-Deficient RPE

**Figure 1:**
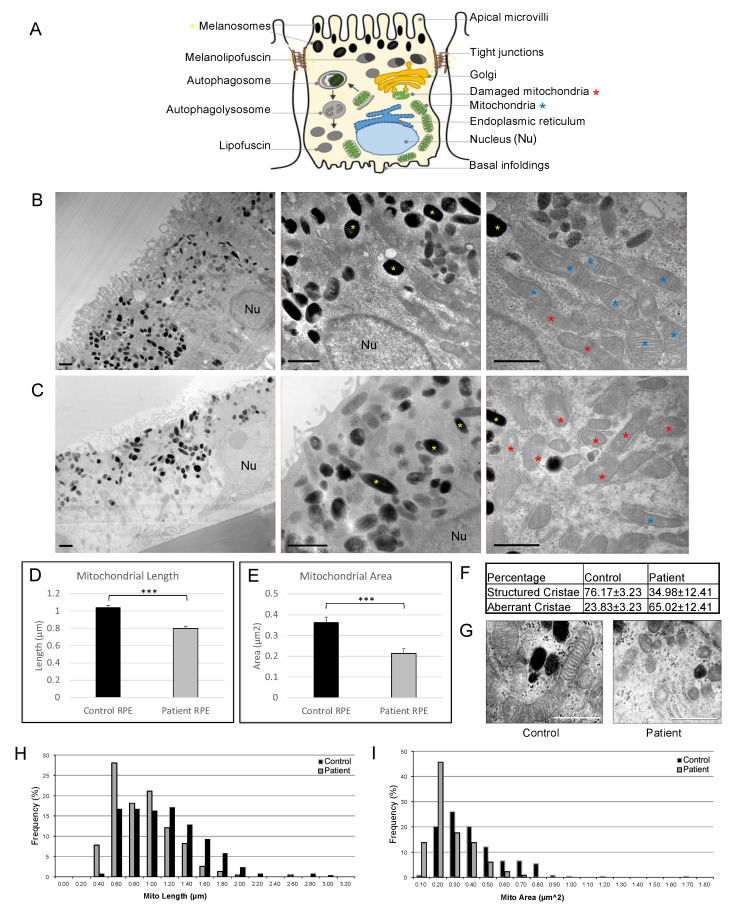
Electron microscopic analysis of cultured RPE cells showing morphology and structural features in patient-derived and control RPE cells. (**A**) A diagram showing the basic structure of RPE cell. (**B**,**C**) TEM images of RPE cells demonstrating the ultrastructure of control-(**B**) and patient-derived (**C**) RPE. Melanosomes (yellow asterisk) were observed in the apical RPE cytoplasm and nuclei (Nu) in the basal cytoplasm. Apical microvilli, tight junctions, endoplasmic reticulum and abundant mitochondria (blue asterisk) were observed. Note the disrupted cristae formation and architecture of damaged mitochondria (red asterisk) in patient-derived RPE. (**D**,**E**) Mean mitochondria length (**D**) and area (**E**) were significantly decreased inpatient RPE cells compared with control groups. (**F**,**G**) An increase in damaged mitochondria with aberrant cristae was observed in patient RPE cells, compared with control RPE cells. (**H**,**I**) Frequency distribution plots of mitochondria by size (**H**) and area (**I**). At least 200 mitochondria were measured in each group. Scale bars in (**B**,**C**,**G**) indicate 1 µm. *** *p* < 0.0001.

Transmission electron microscopic (TEM) images of the control-derived iPSC-RPE monolayers revealed features of native RPE tissue, showing apical microvilli, cytoplasmic melanosomes, nucleus, abundant endoplasmic reticulum and mitochondria. In contrast, patient RPE monolayers appeared more disorganized compared with the control RPE cells. Additionally, mitochondrial abnormalities, including disrupted cristae and a swollen appearance, were observed in patient RPE cells (Figure 1A–C). To further examine mitochondrial ultrastructure in patient RPE cells, mean mitochondrial length was determined by measuring > 200 individual mitochondria from TEM micrographs of RPE cells derived from control and patient iPSC lines. Mitochondria length (Figure 1D) and mitochondria area (Figure 1E) in patient RPE cells (length: 0.79 ± 0.34 µm; area: 0.21 ± 0.12 µm^2^) were significantly decreased compared with control RPE cells (length: 1.04 ± 0.43 µm, *p* < 0.0001; area: 0.37 ± 0.2 µm^2^, *p* < 0.0001). Furthermore, a comparison of the percentages of mitochondria with aberrant cristae revealed increased frequencies of degenerate mitochondria in patient RPE cells (Figure 1F,G). Frequency distribution plots for mitochondrial length and area demonstrated decreased mitochondrial size in patient RPE cells compared with control RPE cells (Figure 1H,I). Representative TEM images illustrating organelle structures in control and patient iPSC-RPE cells are shown in Supplementary Figure S3.

### 3.4. Mitochondrial Membrane Potential Is Reduced in RCBTB1-Deficient RPE

**Figure 2:**
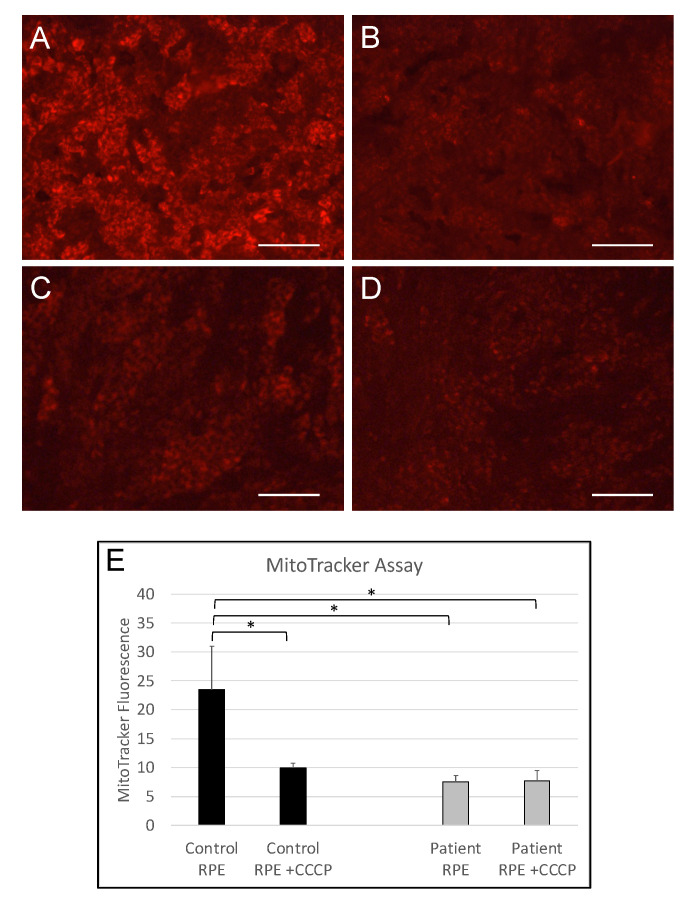
MitoTracker labelling of RPE mitochondria. (**A**–**D**) Fluorescence micrographs showing live control- (**A**,**B**) and patient-derived (**C**,**D**) RPE monolayers labelled with MitoTracker Orange CMTMRos reagent. Panels on the right (**B**,**D**) were treated with 10 μM CCCP. Scale bars indicate 100 μm. (**E**) Bar graph showing mean MitoTracker fluorescence intensities in control- and patient-derived RPE cells, with and without CCCP treatment. Each bar represents the mean Mitotracker signal from RPE monolayers derived from three independent iPSC lines. Error bars indicate standard deviation. * *p* < 0.05.

To further characterize mitochondrial function in iPSC-RPE cells, we incubated the patient and control iPSC-RPE with the MitoTracker Orange CMTMRos probe, which accumulates in mitochondria in a mitochondrial membrane potential-dependent manner. Untreated control iPSC-RPE cells were brightly labelled with the MitoTracker probe, while treatment of RPE cells with the mitochondrial membrane potential disruptor CCCP significantly reduced MitoTracker fluorescence (*p* = 0.0353). In contrast, MitoTracker fluorescence was significantly reduced in untreated patient iPSC-RPE compared with untreated control RPE (*p* = 0.0214) and was unaffected by CCCP treatment (Figure 2), suggesting mitochondrial membrane potential is compromised in RCBTB1-deficient iPSC-RPE.

### 3.5. Reactive Oxygen Species Are Increased in RCBTB1-Deficient RPE

**Figure 3:**
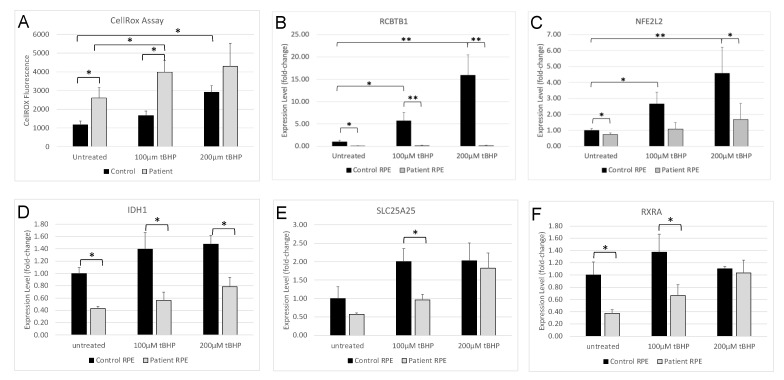
Response of RPE cells to oxidative stress induced by tBHP. Control- and patient-derived RPE monolayers were treated with 0, 100 or 200 μM of tBHP for 1 h. (**A**) ROS levels in live control- and patient-derived RPE monolayers were measured by CellROX assay. (**B–F**) Expression of *RCBTB1* (**B**), *NFE2L2* (**C**), *IDH1* (**D**), *SLC25A25* (**E**) and *RXRA* (**F**) was measured by qPCR. Each bar represents the mean values obtained from RPE cells generated from three independently derived iPSC lines. Error bars indicate standard deviation. * *p* < 0.05; ** *p* < 0.01.

To measure ROS production in iPSC-derived RPE cell cultures, we utilized the CellROX assay (Figure 3A). Untreated patient iPSC-derived RPE cells showed significantly increased levels of ROS compared with iPSC-RPE derived from healthy controls (*p* = 0.015). In control iPSC-RPE cells, treatment with 100 µM tBHP induced a small, non-significant increase in ROS compared with untreated controls (*p* = 0.0544), while treatment with 200 µM induced a larger, significant increase in ROS levels (*p* = 0.0018). In contrast, patient iPSC-RPE showed a large, significant increase in ROS levels in response to 100 µM tBHP compared with untreated patient cells (*p* = 0.0463) or control iPSC-RPE cells treated with 100 µM tBHP (*p* = 0.0037). Treatment of patient iPSC-RPE with 200 µM tBHP resulted in no further increase in ROS levels and were not significantly different from control iPSC-RPE treated at this dose (*p* = 0.1378). Together, these results indicate RCBTB1-deficient RPE cells generate high levels of ROS and, compared to control iPSC-RPE cells, are more sensitive to tBHP-induced oxidative stress.

### 3.6. RCBTB1 Expression Is Induced by Oxidative Stress

Given that *RCBTB1* deficiency has previously been associated with decreased expression of the oxidative stress response gene *NFE2L2* [2], we hypothesized that *RCBTB1* might also be regulated by oxidative stress. To determine if oxidative stress can activate the expression of the *RCBTB1* gene in RPE cells, RNA was harvested from tBHP-treated iPSC-RPE cells and qPCR was performed to detect the expression level of *RCBTB1*. *RCBTB1* mRNA expression was significantly increased in control iPSC-RPE cells treated with either 100 µM (*p* = 0.0142) or 200 µM (*p* = 0.0049) tBHP, compared with untreated control cells. No significant differences in *RCBTB1* expression levels were detected in patient-derived iPSC-RPE cells treated with different concentrations of tBHP (Figure 3B). This result indicates *RCBTB1* expression is responsive to ROS levels in healthy RPE cells.

### 3.7. Oxidative Stress-Induced Activation of NFE2L2 Is Attenuated in Patient RPE

To evaluate the NFE2L2-driven oxidative stress response in RPE cells, the expression level of *NFE2L2* was measured in tBHP-treated RPE cells by qPCR (Figure 3C). *NFE2L2* expression was significantly reduced in untreated patient iPSC-RPE cells compared with untreated control iPSC-RPE cells (*p* = 0.0453). In control iPSC-RPE cells, tBHP treatment significantly increased the expression of *NFE2L2* (*p* = 0.0437 at tBHP, *p* = 0.0012 at 200 µM). In contrast, patient-derived RPE cells showed no significant change in *NFE2L2* expression at either dose. Compared with control RPE, patient RPE cells showed a non-significant reduction in *NFE2L2* expression at 100 µM tBHP (*p* = 0.0622) and a significant reduction in *NFE2L2* expression following treatment with 200 µM tBHP (*p* = 0.0161). The expression of the NFE2L2 target genes *IDH1*, *SLC25A25* and *RXRA* was also reduced inpatient RPE cells compared with controls. Significantly reduced *IDH1* expression was observed in patient RPE cells at all tBHP doses tested (*p* < 0.05). Interestingly, *SLC25A25* and *RXRA* expression showed significant reductions in expression at 100 µM tBHP but were not significantly different from controls at 200 µM tBHP, suggesting NFE2L2-independent upregulation of these genes at high ROS levels. Together, these results demonstrate that oxidative stress-induced activation of the NFE2L2 response is attenuated in patient RPE, suggesting that RCBTB1 may play a role in the NFE2L2-mediated antioxidant responses that protect RPE cells against oxidative stress.

### 3.8. RCBTB1 Interacts with CUL3 and UBE2E3 in RPE Cells

**Figure 4:**
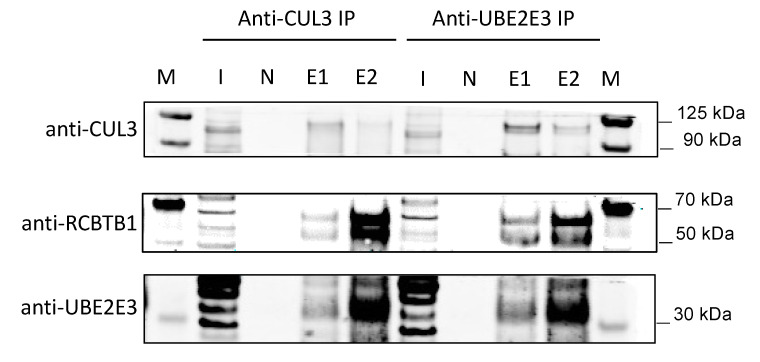
Western blots showing immunoprecipitation results from control iPSC-derived RPE. Protein lysates from control RPE cultures were immunoprecipitated using beads conjugated with either anti-CUL3 (left side) or anti-UBE2E3 (right side) antibodies and analysed by Western blotting using anti-CUL3 (top), anti-RCBTB1 (middle) or anti-UBE2E3 (bottom). Western blot analysis demonstrated coimmunoprecipitation of cullin-3, UBE2E3 and RCBTB1 proteins in RPE cells derived from healthy control iPSC lines using both antibodies. Lanes: M: Protein ladder; I: 10 μg input protein; N: control immunoprecipitation with beads alone (no conjugated antibody, elution 2); E1: Immunoprecipitation with antibody-conjugated beads, elution 1 (without beta-mercaptoethanol); E2: Immunoprecipitation with antibody-conjugated beads, elution 2 (with beta-mercaptoethanol).

To identify potential interactions between RCBTB1 and the CUL3 or UBE2E3 proteins, we performed immunoprecipitation (IP) assays using Sepharose beads conjugated to anti-CUL3 or anti-UBE2E3 antibodies, followed by western blot analysis (Figure 4). RPE cells derived from healthy control iPSC expressed all three proteins, with both UBE2E3 and RCBTB1 showing multiple immunoreactive bands (Figure 4, Input lanes). Following IP with anti-CUL3 beads, cullin-3, UBE2E3 and RCBTB1 proteins were detected by western blot (Figure 4, left side). Interestingly, anti-CUL3 IP pulled down 2/3 UBE2E3 isoforms and 2/4 RCBTB1 isoforms detected in total protein samples, suggesting selective inclusion of specific isoforms in cullin-3 complexes. Similar results were obtained using anti-UBE2E3 conjugated beads, which demonstrated coimmunoprecipitation of cullin-3, UBE2E3 and RCBTB1 proteins (Figure 4, right side). No proteins were detected following IP using unconjugated beads. Together, these results indicate that RCBTB1 is present in protein complexes containing cullin-3 and UBE2E3 in human RPE cells, consistent with its proposed function as a ubiquitin adapter protein [12].

## 4. Discussion

To date, phenotypic features of *RCBTB1*-associated retinopathy have been reported in fifteen cases from eleven families, among which eleven cases from nine unrelated families presented with progressive late-onset macular chorioretinal atrophy and peripheral retinal abnormalities in their 40s or 50s [2,3,5], while four cases manifested a typical retinitis pigmentosa phenotype in their 20s [2,4]. The clinical onset of retinal atrophy in the nine families ranged between 30 and 62 years of age. These patients mostly presented with gradually reduced visual acuity or visual distortion as a result of chorioretinal macular atrophy [3,5]. OCT and fundus autofluorescence (FAF) in patients with *RCBTB1*-associated retinopathy revealed attenuation of the RPE layer and the adjacent ellipsoid zone in the atrophic retinal lesions [3,5]. In light of the available multimodal retinal imaging data showing enlarging RPE atrophic lesions and widespread RPE irregularities, we speculated that the primary defect might occur in the central RPE with centrifugal expansion followed by severe RPE loss and secondary photoreceptor cell loss. To explore this hypothesis, we previously generated iPSC lines from a patient with *RCBTB1*-associated retinopathy and differentiated these into RPE cells [7]. Compared with healthy control RPE, patient RPE cells showed reduced primary cilia lengths, as well as reduced expression of *NFE2L2* and its target genes [8]. In the present study, we extend these observations to demonstrate that levels of ROS are significantly elevated in patient iPSC-derived RPE cells. We further showed that patient-derived RPE cells were more sensitive to tBHP-induced oxidative stress, generating significantly higher ROS than control RPE when treated with the lower dose of tBHP. The increased sensitivity of patient RPE cells to tBHP stimulation was associated with a failure to upregulate expression of the *NFE2L2* gene in response to the oxidative stress challenge. Robust upregulation of *NFE2L2* was observed in control RPE cells in response to tBHP stimulation but not in patient-derived RPE cells. Together, these results indicate that RCBTB1 deficiency is associated with increased oxidative stress and diminished antioxidant responses in RPE cells. In support of these findings, a recent abstract by Carron et al. reported that the knockdown of *RCBTB1* in ARPE19 cells caused changes in cellular responses to exogenous oxidative stress [13].

In a recent report describing three phenotypes of mitochondrial retinopathy, Birtel et al. commented on the resemblance of type 3 mitochondrial retinopathies to *RCBTB1*-associated retinopathy [6]. Here, we observed alterations in mitochondrial ultrastructure in patient-derived RPE by electron microscopy. Compared with healthy control RPE, mitochondria in patient-derived RPE were smaller and displayed disrupted cristae more frequently. Furthermore, mitochondria in patient-derived RPE were poorly labelled by the MitoTracker dye compared with control RPE, consistent with reduced mitochondrial membrane potential [14]. It remains unclear whether the mitochondrial dysfunction observed is a consequence or a cause of the increased ROS levels in RCBTB1-deficient RPE cells; however, this data indicates that damage to RPE mitochondria is a key pathogenic process in RCBTB1-associated retinopathy.

Current understanding of the cellular functions of the RCBTB1 protein is limited, although a number of studies have provided important insights. Using a yeast two-hybrid approach, Plafker et al. demonstrated that the RCBTB1 protein directly interacts with the ubiquitin-conjugating enzyme, UbcM2 (the mouse homologue of the human UBE2E3 protein), suggesting it functions as a CUL3 substrate adaptor in the ubiquitin proteosome system (UPS) [15]. These authors later showed that UbcM2 binds to and regulates the activity of the Nrf2 protein (the mouse homologue of human NFE2L2), a key transcription factor involved in the cellular response to oxidative stress [12]. Under homeostatic conditions, Nrf2 is constitutively ubiquitinated and degraded by the UPS; however, this process is interrupted in response to oxidative stress, leading to the stabilisation of Nrf2 protein and translocation to the nucleus. In the nucleus, Nrf2 binds to the antioxidant response element in the *Nrf2* gene promoter, amplifying its expression in a positive feedback loop. Additionally, Nrf2 activates the expression of network genes involved in the cellular antioxidant defence response [16,17,18,19,20]. Together, these studies suggest the intriguing possibility that RCBTB1 may interact with UBE2E3 to regulate the activation of cellular oxidative stress responses. In support of this hypothesis, our data showed *NFE2L2* expression was unresponsive to increasing ROS levels in patient RPE cells lacking RCBTB1. Furthermore, in healthy control, RPE cells, *RCBTB1* and *NFE2L2,* were both upregulated in a dose-dependent manner in response to increased ROS levels. Inspection of the *RCBTB1* genomic sequence revealed an NFE2L2 antioxidant response element (TGACCCGGC) located 25 base pairs upstream of the putative transcriptional start site in exon 1, suggesting the *RCBTB1* gene is a target for NFE2L2-mediated induction. Additionally, our data showing the coimmunoprecipitation of RCBTB1 and UBE2E3 in human RPE cells provides a direct link between RCBTB1 and NFE2L2 pathway activation.

## 5. Conclusions

In summary, our study provides new insights into the biological role of RCBTB1 in oxidative stress responses and highlights RPE mitochondria as a key target site in the pathogenesis of *RCBTB1*-associated retinopathy. Our results help to explain the similarity in disease phenotype between *RCBTB1*-associated retinopathy and the most severe form of mitochondrial retinopathy. The similarity in pathogenesis between these conditions suggests that therapeutic approaches aimed at alleviating mitochondrial dysfunction may be useful for treating *RCBTB1*-associated retinopathy.

## Data Availability

The data presented in this study are available in the Supplementary Materials.

## Supplementary Materials

The following supporting information can be downloaded at: https://www.mdpi.com/article/10.3390/cells12101358/s1, Supplementary Figure S1: Characterization of iPSC-RPE Cells; Supplementary Figure S2: iPSC-RPE Phagocytosis Assays; Supplementary Figure S3: Electron microscopy of iPSC-RPE cells; Supplementary Figure S4: Original Western Blot Images; Supplementary Table S1: Primer List; Supplementary Table S2: Antibody Information.
