# Supplementary material for: Mitochondrial Dysfunction and Impaired Antioxidant Responses in Retinal Pigment Epithelial Cells Derived from a Patient with RCBTB1-Associated Retinopathy
Source: Cells. 2023 May 10;12(10):1358. doi: 10.3390/cells12101358 (PMC10216830; doi:10.3390/cells12101358)
Supplement: Supplementary file 1 [file cells-12-01358-s001.zip › cells-2345365-supplementary.pdf]

Mitochondrial dysfunction and impaired antioxidant responses in retinal pigment epithelial cells derived from a patient with RCBTB1-associated retinopathy

Zhiqin Huang, Dan Zhang, Shang-Chih Chen, David Mackey, Fred K. Chen, Samuel McLenachan

SUPPLEMENTARY MATERIALS

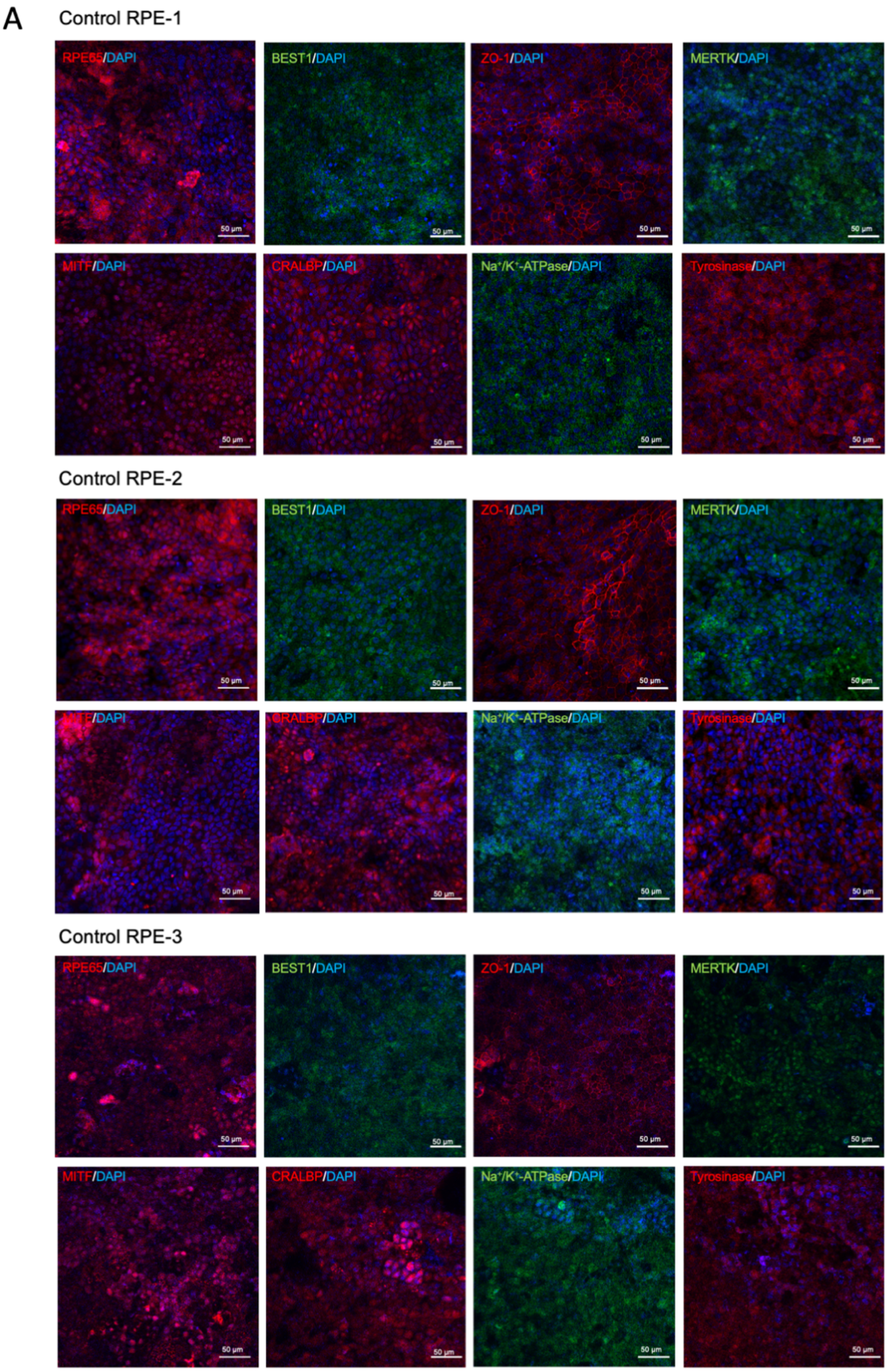
A
Control RPE-1
RPE65/DAPI
BEST1/DAPI
ZO-1/DAPI
MERTK/DAPI
MITF/DAPI
CRALBP/DAPI
Na+/K+-ATPase/DAPI
Tyrosinase/DAPI
Control RPE-2
RPE65/DAPI
BEST1/DAPI
ZO-1/DAPI
MERTK/DAPI
MITF/DAPI
CRALBP/DAPI
Na+/K+-ATPase/DAPI
Tyrosinase/DAPI
Control RPE-3
RPE65/DAPI
BEST1/DAPI
ZO-1/DAPI
MERTK/DAPI
MITF/DAPI
CRALBP/DAPI
Na+/K+-ATPase/DAPI
Tyrosinase/DAPI
50 μm

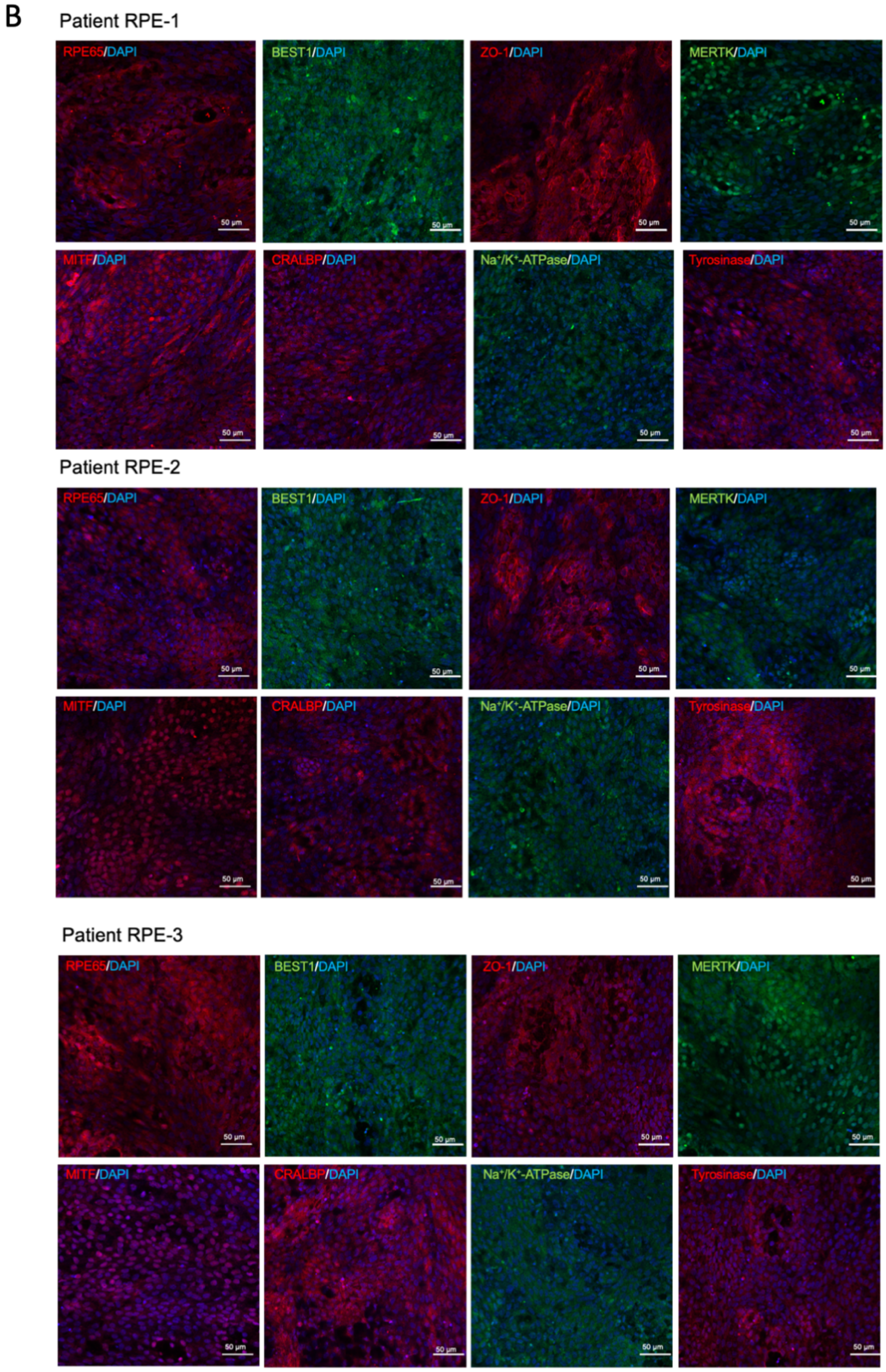
B
Patient RPE-1
RPE65/DAPI
BEST1/DAPI
ZO-1/DAPI
MERTK/DAPI
MITF/DAPI
CRALBP/DAPI
Na+/K+-ATPase/DAPI
Tyrosinase/DAPI
Patient RPE-2
RPE65/DAPI
BEST1/DAPI
ZO-1/DAPI
MERTK/DAPI
MITF/DAPI
CRALBP/DAPI
Na+/K+-ATPase/DAPI
Tyrosinase/DAPI
Patient RPE-3
RPE65/DAPI
BEST1/DAPI
ZO-1/DAPI
MERTK/DAPI
MITF/DAPI
CRALBP/DAPI
Na+/K+-ATPase/DAPI
Tyrosinase/DAPI

C

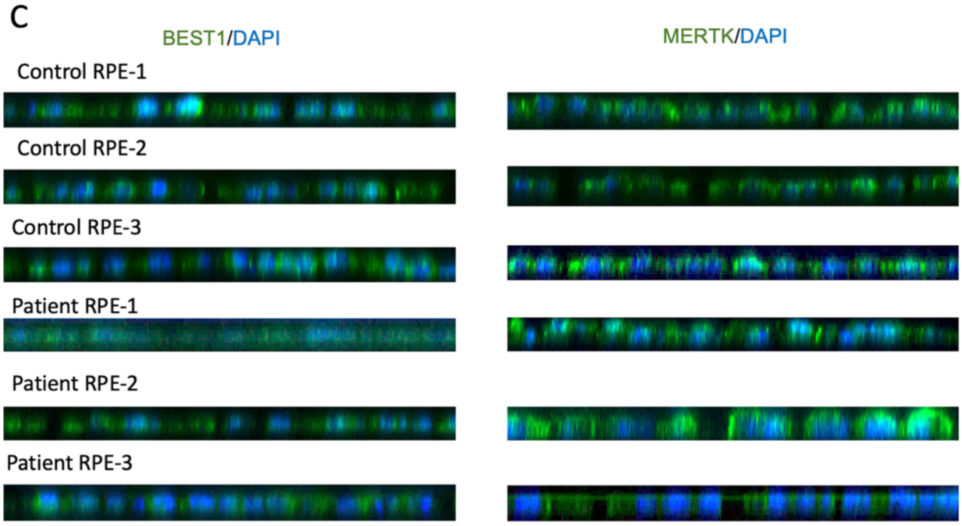

D

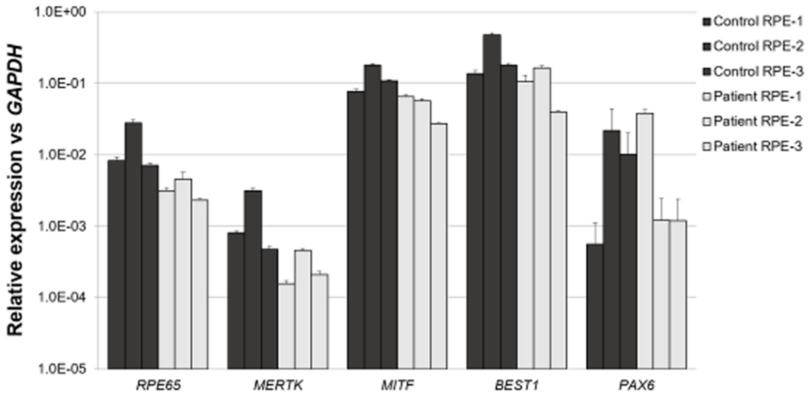

Supplementary Figure S1: A-B: RPE cells were derived from three iPSC lines generated from two healthy control subjects (A) and three clonal iPSC lines derived from a patient with RCBTB1-associated retinopathy (B). RPE monolayers were fixed for immunostaining six weeks after plating. The identity of iPSC-RPE cells was confirmed by immunostaining for the RPE markers RPE65, bestrophin1, ZO1, MERTK, MITF, CRALBP, Na+/K+ ATPase and Tyrosinase (red and green signals). Nuclei were stained with DAPI (blue signal). RPE derived from all six iPSC lines expressed all RPE markers tested. **C:** Side views of confocal z-stacks (apical side up) showed basal localization of bestrophin and apical localization of MerTK in control and patient RPE cells. **D:** Gene expression was measured by qRT-PCR in RPE monolayers derived from three patient-iPSC lines and three control iPSC lines, six weeks after seeding. Bars indicate mean *RPE65*, *MERTK*, *MITF*, *BEST1* and *PAX6* gene expression values normalized to *GAPDH*. Error bars show standard deviation.

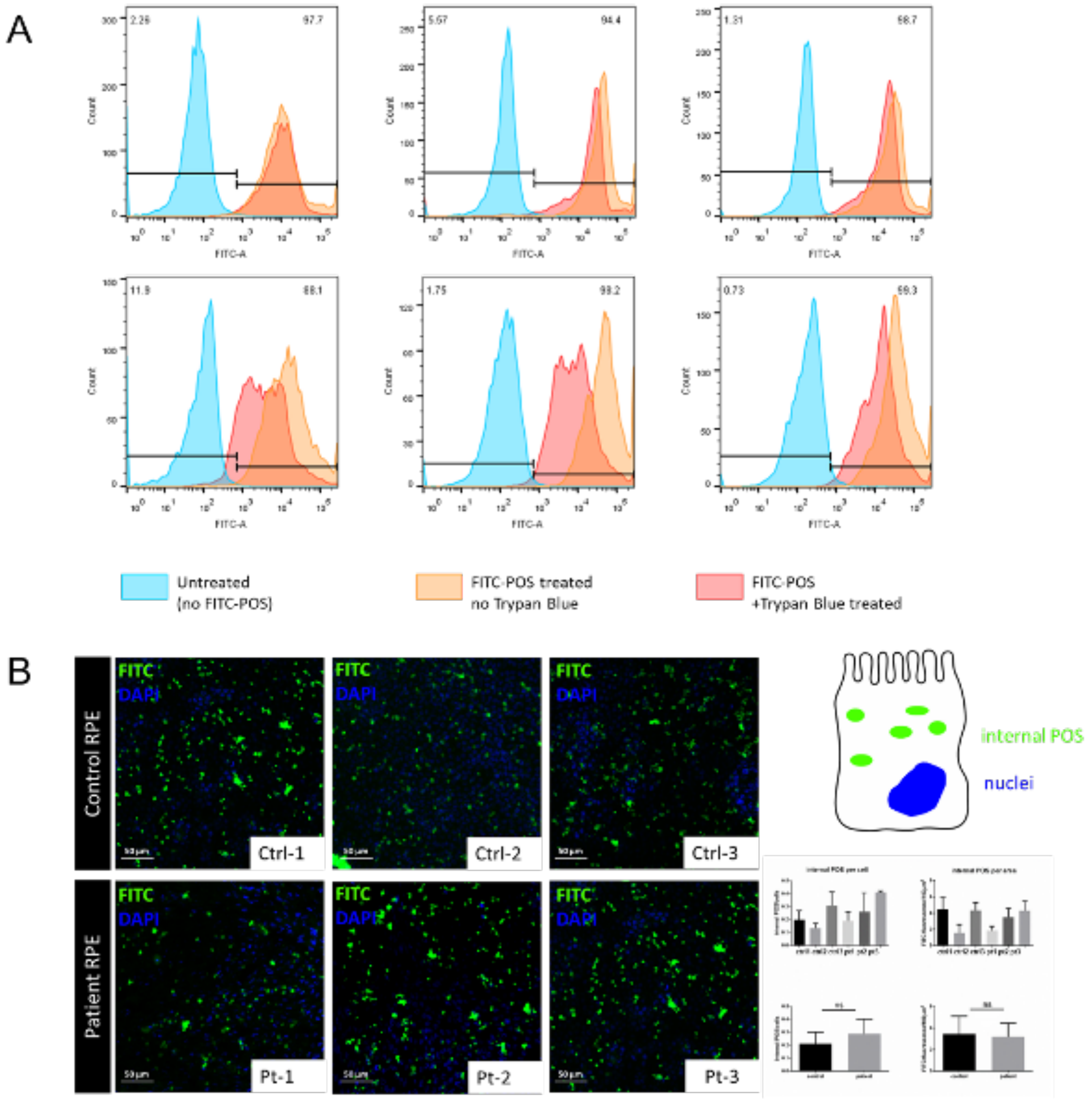

Supplementary Figure S2: A) Flow cytometry data presented as histograms showing quantification of control and patient RPE phagocytosis of FITC-labelled photoreceptor outer segment (POS), which indicated slightly less active POS uptake by patient RPE-1 cells than other patient RPE and control derived RPE. Overlay of untreated population onto the stained population allows identification of RPE cells with internalized or surface-bound FITC-POS. Percentage of cells internalizing POS was indicated in the top left (FITC negative) and top right (FITC positive) in each histogram. **B)** Merged micrographs demonstrating phagocytized

FITC-labelled POS in control and patient derived RPE cells. No significant difference was observed with regards to internalized POS amount among the control and patient RPE groups. Error bars indicate standard deviation.

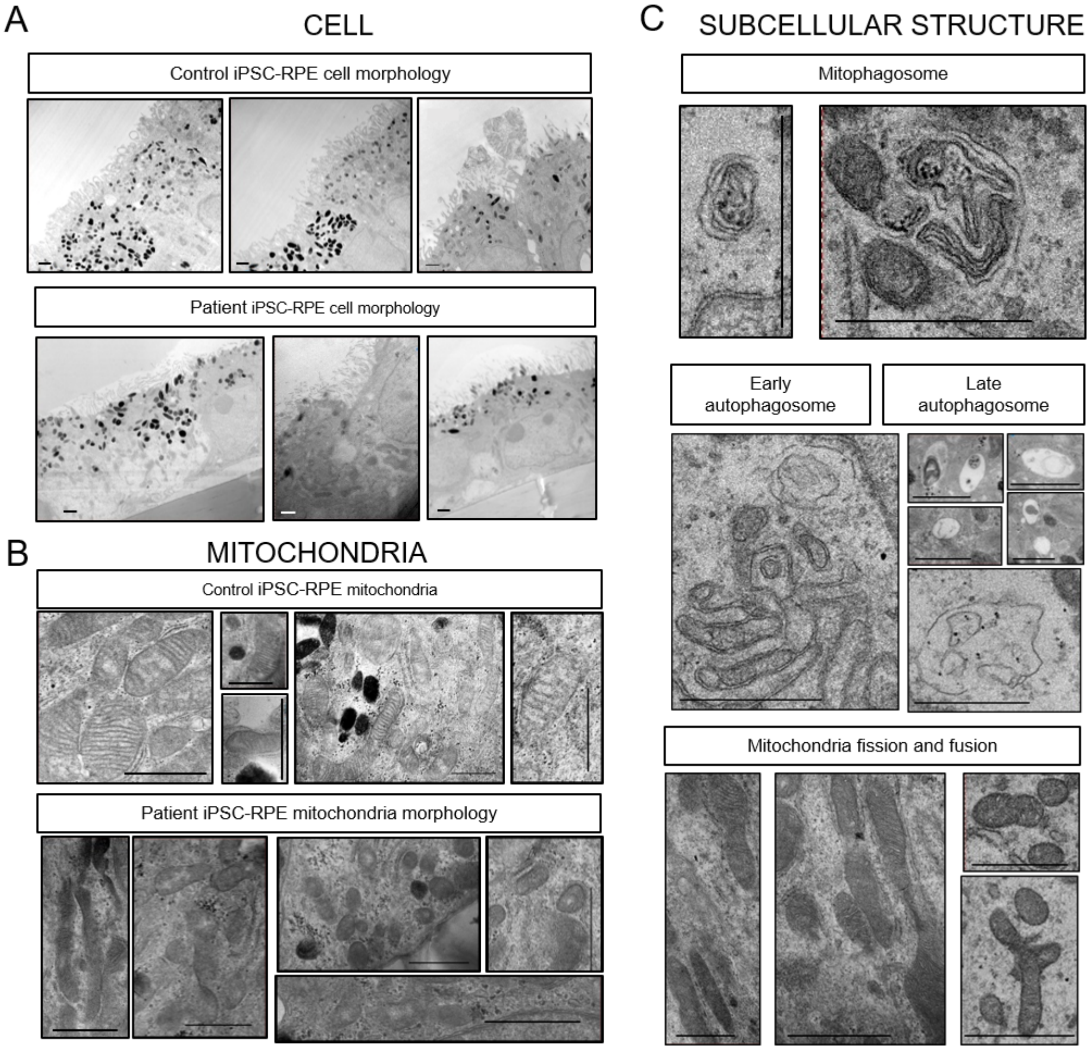

Supplementary Figure S3: Representative transmission electron microscopy micrographs of iPSC-RPE cells. Scale bar = 1 µm. **A)** Representative TEM images of control and patient derived iPSC-RPE cells. **B)** Representative TEM micrographs of mitochondria in control and patient derived iPSC-RPE cells. **C)** Representative TEM micrographs showing subcellular organelles including mitophagosome, early autophagosome and late autophagosome. Mitochondrial fusion and fission events were observed in iPSC-RPE cells.

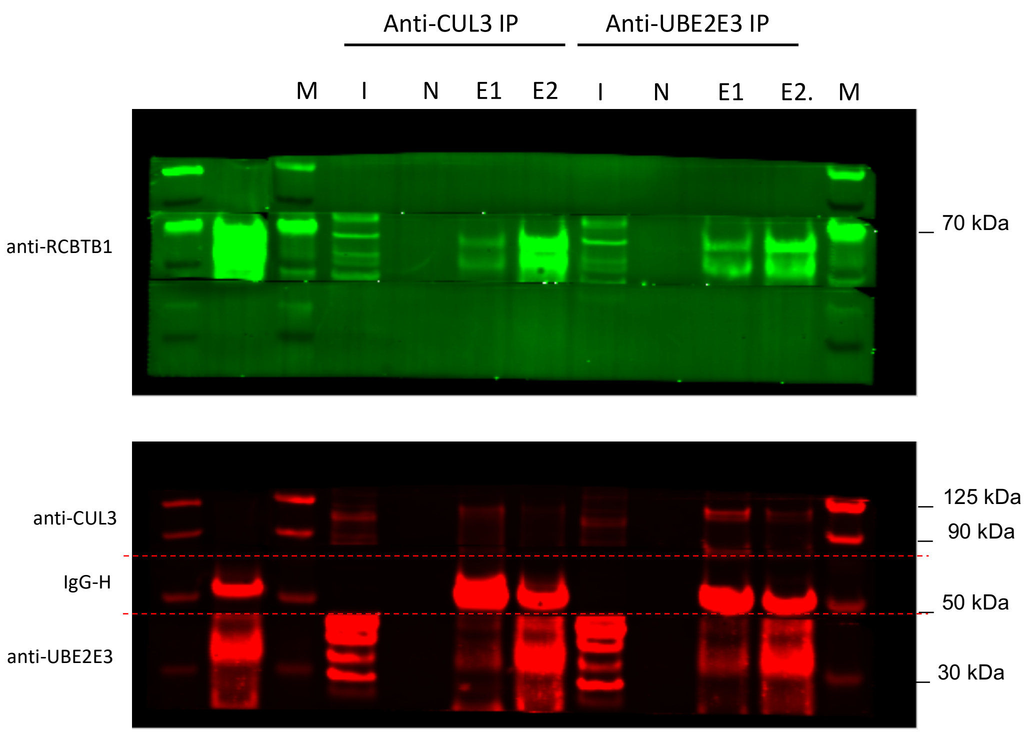

Supplementary Figure S4: Original western blot images shown in Figure 4. Lanes 1 and 2 contain Protein Marker Ladder and human retinal protein lysate, respectively.

Supplementary Table S1. Primer list

**Target**	**Forward/Reverse primer (5′–3′)**
RPE65	TTTGGCACCTGTGCTTTCCCAG/GTTGGTCTCTGTGCAAGCGTAG
MERTK	AGACTTCAGCCACCCAAATG/GGGCAATATCCACCATGAAC
MITF	GGCTTGATGGATCCTGCTTTGC/GAAGGTTGGCTGGACAGGAGTT
BEST1	TGCCAACCTGTCAATGAAGGCG/TCCAGTCGTAGGCATACAGGTG
PAX6	AACGATAACATACCAAGCGTGT/GGTCTGCCCGTTCAACATC
RCBTB1	TCCTGAGCTCTGTAGTTGAAATG/CAGAGTTAGCCCGAAGTGTTTA
NFE2L2	GTTGCCCACATTCCCAAATC/CGTAGCCGAAGAAACCTCAT
RXRA	GACGGAGCTTGTGTCCAAGAT/AGTCAGGGTTAAAGAGGACGAT
IDH1	AGAAGCATAATGTTGGCGTCA/CGTATGGTGCCATTTGGTGATT
SLC25A25	AGAATGATGGACGCATTGAC/ATGGTCATCGTGCCGTTT
GAPDH	GTCTCCTCTGACTTCAACAGCG/ACCACCCTGTTGCTGTAGCCAA

Supplementary Table S2. Antibody information

**Antibodies used for immunocytochemistry**			
	**Antibody**	**Dilution**	**Company Cat # and RRID**
RCBTB1	Rabbit anti-RCBTB1	1/500	Thermo Fisher Scientific Cat# PA5-30672, RRID:AB_2548146
RPE markers	Mouse anti-RPE65	1/200	Santa Cruz Cat# sc-390787
	Rabbit anti-BEST1	1/200	Abcam Cat# ab14928, RRID: AB_301519
	Mouse anti-ZO-1	1/100	Invitrogen Cat#339194, RRID: AB_2533147
	Rabbit anti-MERTK	1/100	Abcam Cat# ab52968, RRID: AB_2143584
	Mouse anti-MITF	1/200	Invitrogen Cat#MA5-14154, RRID: AB_10982126
	Mouse anti-CRALBP	1/200	Abcam Cat# ab15051, RRID: AB_2269474
	Rabbit anti-Na^+/K^+-ATPase	1/100	Cell signalling #3010, RRID: AB_2060983
	Mouse anti-Tryosinase	1/100	Abcam Cat#738,RRID: AB_305899
Secondary antibodies	Alexa Fluor 488 Goat anti-rabbit	1/500	Thermo Fisher Scientific Cat# A-11008, RRID:AB_143165
	Alexa Fluor 546 Goat anti-mouse	1/500	Thermo Fisher Scientific Cat# A-11003, RRID:AB_2534071
**Antibodies used for western blotting and immunoprecipitation**			
	**Antibody**	**Dilution**	**Company Cat # and RRID**
RCBTB1	Rabbit anti-RCBTB1	1/500	Thermo Fisher Scientific Cat# PA5-30672, RRID:AB_2548146
Ubiquitin System proteins	Mouse anti-CUL3	1/500	Santa Cruz Biotechnology Cat# sc-166110, RRID:AB_2245478
	Mouse anti-UBE2E3	1/200	Thermo Fisher Scientific Cat# MA5-26364, RRID:AB_2723755
House-Keeping proteins	Mouse anti-GAPDH	1/500	Abcam Cat# ab125247, RRID:AB_11129118
	Mouse anti β-Actin	1/1000	Sigma-Aldrich Cat# A5441, RRID:AB_476744
